# GPUmotif: An Ultra-Fast and Energy-Efficient Motif Analysis Program Using Graphics Processing Units

**DOI:** 10.1371/journal.pone.0036865

**Published:** 2012-05-25

**Authors:** Pooya Zandevakili, Ming Hu, Zhaohui Qin

**Affiliations:** 1 Computer Science and Engineering Department, University of Michigan, Ann Arbor, Michigan, United States of America; 2 Department of Statistics, Harvard University, Cambridge, Massachusetts, United States of America; 3 Department of Biostatistics and Bioinformatics, Emory University, Atlanta, Georgia, United States of America; 4 Center for Comprehensive Informatics, Emory University, Atlanta, Georgia, United States of America; 5 Department of Biomedical Informatics, Emory University, Atlanta, Georgia, United States of America; University of Iowa, United States of America

## Abstract

Computational detection of TF binding patterns has become an indispensable tool in functional genomics research. With the rapid advance of new sequencing technologies, large amounts of protein-DNA interaction data have been produced. Analyzing this data can provide substantial insight into the mechanisms of transcriptional regulation. However, the massive amount of sequence data presents daunting challenges. In our previous work, we have developed a novel algorithm called Hybrid Motif Sampler (HMS) that enables more scalable and accurate motif analysis. Despite much improvement, HMS is still time-consuming due to the requirement to calculate matching probabilities position-by-position. Using the NVIDIA CUDA toolkit, we developed a graphics processing unit (GPU)-accelerated motif analysis program named GPUmotif. We proposed a “fragmentation" technique to hide data transfer time between memories. Performance comparison studies showed that commonly-used model-based motif scan and *de novo* motif finding procedures such as HMS can be dramatically accelerated when running GPUmotif on NVIDIA graphics cards. As a result, energy consumption can also be greatly reduced when running motif analysis using GPUmotif. The GPUmotif program is freely available at http://sourceforge.net/projects/gpumotif/

## Introduction

Accurately locating the transcription factor (TF)-DNA interaction sites provides insight into the underlying mechanisms of transcriptional regulation. Since binding sites for most TFs show sequence specificity, computational prediction of TF binding sites based on such sequence features has demonstrated to be an effective tool for functional genomics research.

New technologies such as ChIP-Seq, or chromatin immunoprecipitation followed by high-throughput sequencing [1], [2], [3], [4], are capable of producing large amounts of sequence data that is believed to harbor protein-DNA binding sites. Motif analyses including known motif scan and *de novo* motif finding are effective tools to help us understand the underlying transcription regulation mechanisms [5]. A *de novo* motif search is helpful even in cases where the TF binding pattern is known since it can reassure the accuracy of data, especially in the common case where these patterns are reported based on limited experimentally verified TF-DNA interaction sites.

In our previous work, we discussed the limitations of current methods and proposed a new *de novo* motif finding algorithm named Hybrid Motif Sampler (HMS) [6]. HMS is specifically designed for analyzing the massive volume of ChIP-Seq data. Because HMS is a probability model-based method, which relies on parameter-rich position-specific weight matrices (PSWM) to characterize motif patterns, despite much improvement, HMS is still time-consuming due to the requirement to calculate matching probabilities position-by-position for every sequence through an iterative process.

Recently, advanced parallel computing hardware such as graphics processing units (GPUs), have greatly enabled massively parallel processing on a desktop computer. Originally designed to accelerate demanding 3D graphics, the power of GPUs has been harnessed for non-graphical, general purpose applications including bioinformatics [7], [8], [9], [10], [11], [12], [13], [14]. For applications containing a very large number of homogeneous tasks that can (almost) be done independently, GPUs, which are classified as “fine-grain" parallel hardware, offer lower cost, less system complexity and better energy efficiency when compared to their “coarse-grain" counter-parts such as many-core architectures and computer clusters. This motivates us to develop a suite of motif analysis programs taking advantage of the powerful GPU.

## Methods

We have developed a software package named GPUmotif that is capable of performing ultra-fast motif analysis. GPUmotif is written in C++ and CUDA C and works on any CUDA-enabled GPU.

Our design is driven by the observation that motif scan constitutes the main portion of the HMS's runtime. As mentioned earlier, although PSWMs provide an effective way to represent the sequence features of TF binding sites, scanning a large number of sequences using PSWM is time-consuming since a matching probability needs to be calculated for each possible start position of every sequence. Thus, we aimed to eliminate this computation bottleneck in model-based motif analysis algorithms such as HMS.

In the following subsections, we first state the statistical models that GPUmotif is based upon, and then proceed to discuss how we use GPU-computing to significantly accelerate motif scan procedure and finally show how we employ this new motif scan core to improve HMS.

### Motif scan

In motif scan, our task is to scan through a series of DNA sequences using a set of known PSWMs, such that given a significance threshold, we are able to report the number of motif incidences for each PSWM. The motif scan core receives the input sequences and PSWMs and outputs the corresponding matching probabilities.

#### Statistical model

Let 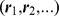 denote a set of DNA sequences, *a* represent the motif start location, and 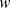 stand for the motif width and is assumed to be known. Let 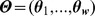 with each 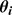 being a probability vector of length four that represents the nucleotide preference at the 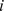 ^th^ position of the motif. For notational simplicity, we use integers 1, 2, 3 and 4 to represent the four types of nucleotides A, C, G and T. *P(Background_t_)* is the background noise calculated using a third-order Markov model as
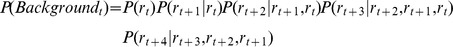


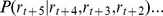



The posterior probability of the corresponding motif starting at each position is calculated for all sequences using the following formula:
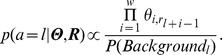
Here 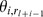 is one of the four parameters in the *i* ^th^ multinomial distribution parameter vector 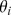 that corresponds to the base found at 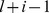.

### *De novo* motif finding

*De novo* motif finding requires no prior knowledge of the TF binding sites. It is designed to delineate over-represented motif patterns from a set of DNA sequences. A variety of different software programs have been developed for motif-finding [15], [16], [17], [18], [19], [20], [21]. See Tompa et al. [22] for a review of this topic.

#### Statistical model

The HMS algorithm [6] is specially designed to analyze massive sequence data produced from high-throughput assays such as ChIP-Seq. HMS is based on the product multinomial model [23] which is commonly used for Gibbs sampler-based algorithms [16], [17], [19]. In order to process tens of thousands of input sequences, HMS implements a prioritized hybrid Monte Carlo strategy that allows us to rapidly analyze a large amount of input sequences and produce an accurate estimate of the motif pattern. In addition, HMS leverages sequencing depth within each region to aid motif identification. For the sake of completeness, we briefly summarize the statistical model introduced in our earlier work.

Let 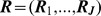 denote a set of 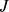 DNA sequences, obtained from ChIP-enriched regions, of lengths 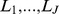 respectively. Let 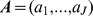 denote the vector that is formed by the start locations with 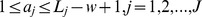. Here 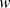 is the motif width and is assumed to be known. Let 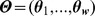 with 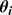 representing the nucleotide preference at the 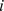 ^th^ position of the motif and let the probability vector 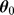 represent the nucleotide preference for non-motif positions in these sequences. Each of the 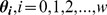 is a probability vector of length four. For notational simplicity, we use integers 1, 2, 3 and 4 to represent the four types of nucleotides, A, C, G and T.

To increase specificity, we introduce a binary indicator variable 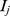 where 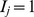 indicates that 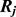 contains at least one motif, and 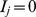 otherwise. In the algorithm, 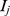 is set to 1 if the average of likelihood ratios observing the motif in the sequence 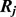, denoted as 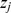, is greater than 1. i.e.,
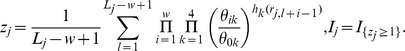
The functions 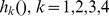 (represents nucleotide A, C, G and T respectively) return the number (0 or 1) of nucleotides of type *k*.

After updating 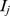, we only conduct motif search on the sequences with 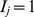.

The probability of background sequences is calculated as before. And the prior probability 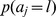 is derived from a student-*t*'s distribution which mimics the sequence depth within the peak.

The parameter of main interest in this model is the alignment variable 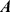. HMS employs a Gibbs sampler type approach as in the original Gibbs motif sampler [17]. Using a conjugate prior for each 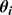, which is *Dirichlet* 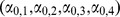, the posterior distribution for alignment 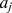 can be expressed as:
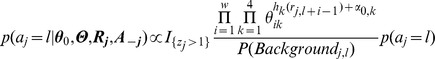



### GPU-accelerated Implementation

During both motif scan and *de novo* motif finding procedures, calculating the posterior probability that a motif starts from a given position is the most computation-intensive step. These probability calculations can be done simultaneously. Therefore, we offload this task to the GPU to take advantage of its concurrent computation capabilities.

**Figure 1:**
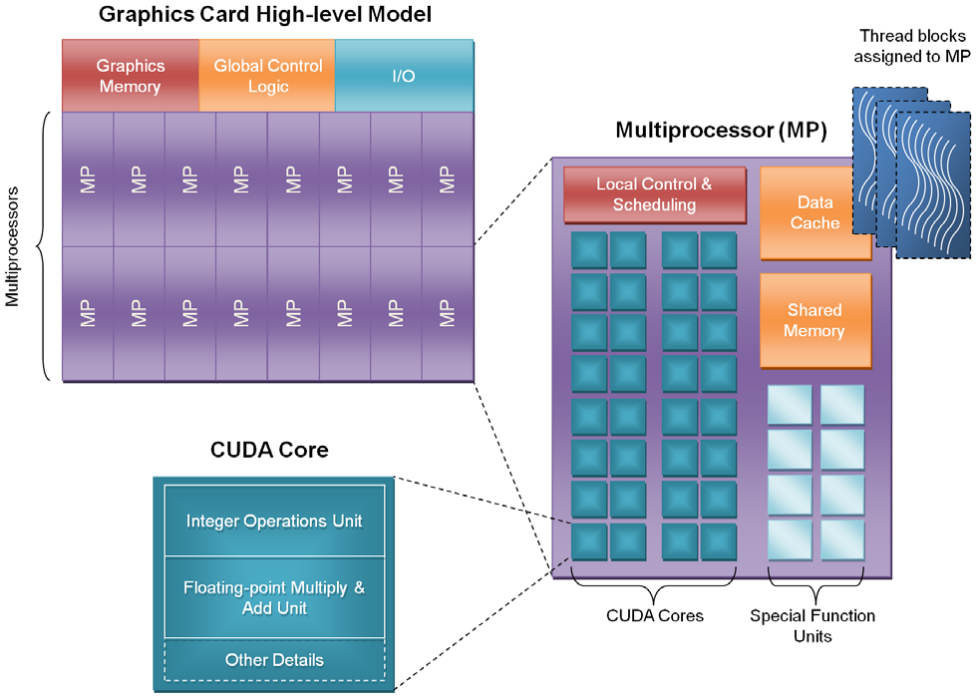
High-level architecture of the NVIDIA GTX 480 graphics card (simplified and excluding the graphics-specific details). The GPU contains several multiprocessors (MPs) for executing groups of threads, called “thread blocks", assigned to MPs by the global control logic (scheduler). Each MP contains several (2×16 for GTX 480) CUDA cores capable of performing floating-point multiply-and-add and integer, logical and bitwise operations. MPs also contain a fast programmable shared memory, hardware-managed data caches and “special function" units that perform double-precision or more complex floating-point operation (such as reciprocal, square root, sine and cosign). A local scheduler assigns resources (including CUDA cores) to the thread blocks assigned to the MP.

Figure 1 shows the high-level architecture of a CUDA-enabled NVIDIA graphics card (GTX 480). We have eliminated graphics-specific details (such as texture units) for more clarity. Computational tasks written for the GPU, “kernels", usually consist of many units of work, called “threads", that can run concurrently. To execute, logical groups of threads, called “thread blocks", are assigned by the global scheduler to the multiprocessors (MPs), simple processing cores that contain several CUDA cores (usually 8 or 32) for concurrent execution of threads and memory resources to accommodate and enhance the storage needs of the threads during execution. Although thread blocks remain on the same MP for their entire lifetime (until all their threads complete), multiple thread blocks can be assigned to the same MP at any given moment, giving the scheduler more flexibility to avoid stalls created by data dependencies and the memory access latency while allowing the threads in the same block to share data and communicate. A local (warp) scheduler inside the MP decides which thread block and which threads inside that thread block (usually groups of 16, called warps) execute during each computational cycle. This execution model, called Single Instruction Multiple Threads, enables extremely high-throughput execution of concurrent units of work (threads), while allowing for more control-flow flexibility than its traditional counter-part, Single Instruction Multiple Data (SIMD). For more detailed information on the CUDA architecture, please see NVIDIA CUDA C Programming Guide, version 4.1. http://developer.download.nvidia.com/compute/DevZone/docs/html/C/doc/CUDA_C_Programming_Guide.pdf.

A typical computational task on a GPU consists of three major steps: first, input-transfer: transferring input data from computer's main memory to the graphics card memory; second, computation: performing the computation on the GPU; third, results-transfer: transferring the results back from the graphics card memory to the main memory.

In the GPUmotif motif scan core, the GPU is setup by first transferring the input sequences and corresponding background sequence information to the graphics card's memory. The input-transfer step is performed only once during the whole lifetime of the program and thus the overhead from this step is negligible. We describe the second and third steps in detail.

Once the card has been setup, we calculate the probabilities for each PSWM. During this step, the actual computation takes place and this is, in fact, where the power of the GPU is harnessed. Because the probabilities evaluated at different starting positions are independent of each other, our problem can be broken down into parallel units of labor, or “threads" as mentioned earlier, with thread *t* calculating the probability that a motif starts at position *t*.

Generally speaking, more threads lead to a more efficient utilization of the GPU and thus higher gains. For this reason, instead of waiting for each sequence to finish and then moving on to the next, we concatenate all the sequences and scan them all at once, as if they were one long sequence. A later post-processing step separates the results for different sequences.

#### Results Transfer

When the probability calculation for all sequences is completed, before moving on to the next PSWM, we have to perform an important step: results-transfer. The results of a GPU task (“kernel") are placed in the graphics card memory. To make these results accessible to the user, they have to be transferred back to the computer's main memory.

**Figure 2:**
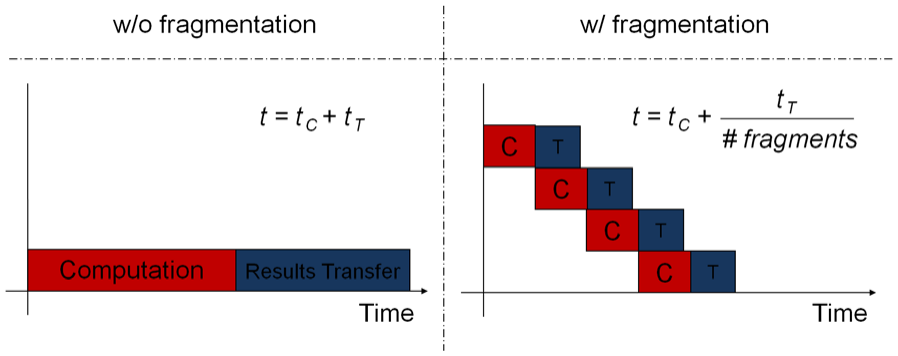
Illustration of the “fragmentation" technique we proposed for improving the performance of GPU.

One of the most common restraining bottlenecks in GPU-accelerated computing is the latency of data transfers between the graphics card memory and the computer's main memory. In this work, we developed a novel and effective technique, named “fragmentation" that virtually eliminates this transfer overhead by shifting it forward and making it happen concurrently with the computation. Taking advantage of the new NVIDIA concurrent transfer capabilities, we break down our scan task into n sub-tasks, or “fragments". The results from fragment *k* are transferred back to the host memory at the same time fragment *k+1* is executing. This almost completely hides the data-transfer overhead thus boosting the performance up to 1.6 times in the motif scan procedure. Figure 2 shows how fragmentation works. It should be noted that this is for the case where computation time is greater than transfer time. In the converse scenario, fragmentation is still helpful but becomes less effective as the ratio of transfer time to computation time grows.

#### Datasets for performance comparison

We integrated our new motif scan core into HMS. To test its performance, we used four publically-available ChIP-Seq datasets: NRSF (neuron-restrictive silencer factor) [1], STAT1 (signal transducer and activator of transcription protein 1) [3], CTCF (CCCTC-binding factor) [2] and ER (estrogen receptor) [6]. We used HPeak [24] to define read-enriched regions from the ChIP-Seq data and used HMS [8] to perform non-GPU *de novo* motif finding.

### Speedup Evaluation

Given the inherent granularity limitations of the conventional timer implementations, to ensure accuracy, all our experiments were performed multiple times and the per-experiment time was derived by dividing the aggregate time by the number of repetitions. Also the data transfer times were counted in for the calculations making each per-experiment time include both the computation and the data-transfer time to achieve a fair comparison.

## Results and Discussion

We compared the computation time of GPUmotif against its non-GPU version using real sequence data. Because the execution time of both the CPU and GPU versions only depend on the size of the input and not the specific sequences, for the performance comparisons in this study, DNA sequences were randomly selected from the reference Human genome sequence. Position-specific weight matrices of motifs with different widths were selected from TRANSFAC [25]. All performance comparisons in this study were conducted on a desktop computer with an Intel Core i7 920 processor and 4 GB of RAM running 64-bit Ubuntu Linux 9.04. Only a single CPU core of the desktop computer was used when testing the non-GPU version. We tested on two different graphics cards, NVIDIA GeForce GTX 260 and the NVIDIA GeForce GTX 480 (Fermi) for a more thorough analysis of the motif scan core performance improvement. For analysis of *de novo* motif search performance, however, we performed our experiments only on the latter.

**Figure 3:**
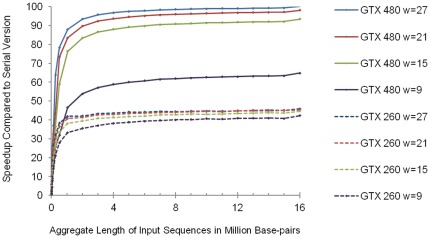
The speedup of the GPU-accelerated motif search over the original version. “w" stands for motif width.

Figure 3 shows the motif scan core speedup results for both cards. As the figure shows, we can achieve speedup as high as 100 times. The increased number of cores and the more advanced architecture of the new Fermi card yield higher speedup.

As the figure shows, longer motifs and longer aggregate input sequences result in further speedup. This is because when the size of the motif increases, the fraction of the program time spent in the scanning loop becomes more salient as compared to scheduling and setup overheads. Also, longer motifs benefit more from fragmentation. Similarly, larger aggregate sequence size, i.e. having longer or simply more input sequences, results in more positions requiring assessment of matching scores. This, in turn, causes an increase in the degree of available concurrency resulting in better utilization of the GPU and also increased freedom of the GPU scheduler in assigning the scan tasks to its computational units. Therefore, more speedup is achieved. Note that the individual sequence lengths do not affect the speedup since all sequences are concatenated before scanning in our algorithm.

Table 1 shows the results for the four ChIP-Seq datasets. The large difference between the speedup of the motif scan core and the overall algorithm might be surprising at first. This difference is, in fact, due to a common phenomenon in parallelization of algorithms referred to as Amdahl's law [26]. Amdahl's law states that the maximum speedup that can be achieved by parallelizing a portion of a program that constitutes fraction *f* of the overall program execution time is 1/(1−*f*). To justify Amdahl's law, imagine a program spends *t_R_* of its overall execution time, *t*, in a specific routine *R*. By breaking *R* into *n* parallel sub-routines, the program time reduces to 
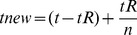
 resulting in a speedup of 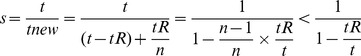. Setting 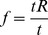 yields Amdahl's law.

**Table 1:** *De novo* search speedup across various datasets.

Dataset	Dataset Size (MB)	Original Time (sec.)	GPU-Aided Time (sec.)	Overall Speedup	% time in Motif Scan Core	Theoretical Speedup Limit
CTCF	7.36	527.5	55.42	9.52	90.62	10.66
ER	2.48	179.36	17.05	10.52	91.80	12.20
NRSF	1.21	84.54	6.82	12.40	93.46	15.29
STAT1	6.77	519.26	72.82	7.13	87.13	7.77

Here, the portion of the program that has been parallelized (R) is the motif scan core. The fraction time that the HMS spends in the motif scan core, depends on the input dataset (by a few percents) and has been shown for each dataset in Table 1 (“*f*" is determined using a tool called the GNU profiler (gprof) that outputs the amount of time spent in each function of a running program).

For instance, for CTCF, HMS spends 90.62% of its execution time in the motif scan core and thus by Amdahl's law, the theoretical upper bound for speedup (through parallelizing the motif scan core) is 
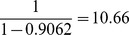
. As the table shows, our implementation does get very close to this maximum.

### Energy consumption comparison

Although graphics cards draw additional power when active, utilizing them significantly reduces the computation time for a given task therefore leading to improved energy-efficiency. In the case of our motif scan core, the original non-GPU version draws 324 Joules (measured by a digital meter) to scan a 21 bp motif on a 16-MB sequence. The same scan draws only 12.8 Joules on GTX 260 and 7.6 Joules on GTX 480 which is an improvement by a factor of 25 and 42 times respectively. Our findings indicated that mundane bioinformatics jobs such as motif scan and discovery can benefits from the latest GPU-computing technology, achieving not only dramatic speedup in computing time, but also significant savings in energy consumption.
